# MHC class I loci of the Bar-Headed goose (*Anser indicus*)

**DOI:** 10.1590/S1415-47572010000300031

**Published:** 2010-09-01

**Authors:** Qinglong Liang, Lei Wei, Xinwei Wang, Hongxuan He

**Affiliations:** 1 Key Laboratory of Animal Ecology and Conservation Biology, Institute of Zoology, National Research Center For Wildlife-Borne Diseases, Chinese Academy of Sciences, BeijingChina; 2 Graduate University, Chinese Academy of Sciences, BeijingChina

**Keywords:** MHC I, *Anser indicus*, polymorphism, phylogenetic analysis

## Abstract

MHC class I proteins mediate functions in anti-pathogen defense. MHC diversity has already been investigated by many studies in model avian species, but here we chose the bar-headed goose, a worldwide migrant bird, as a non-model avian species. Sequences from exons encoding the peptide-binding region (PBR) of MHC class I molecules were isolated from liver genomic DNA, to investigate variation in these genes. These are the first MHC class I partial sequences of the bar-headed goose to be reported. A preliminary analysis suggests the presence of at least four MHC class I genes, which share great similarity with those of the goose and duck. A phylogenetic analysis of bar-headed goose, goose and duck MHC class I sequences using the NJ method supports the idea that they all cluster within the anseriforms clade.

The major histocompatibility complex (MHC) is a gene complex encoding the receptors which bind endogenous or exogenous antigenic peptides for presentation to cytotoxic T-lymphocytes, an important step for the initiation of the majority of immune responses in vertebrates. Much of the polymorphism in the MHC class I occurs in the regions encoded by exons 2 and 3, which bind the peptides to create variation in binding specificity. This polymorphism is considered important, both for handling a wide range of evolving pathogens and for the health and conservation of species. The bar-headed goose (*Anser indicus*) breeds in Central Asia and migrates southward over the Himalayas to India or northern Burma for the winter.

We chose to study the MHC class I of this species because it may provide relevant information regarding viral disease sensitivity in this group of birds. Successful cloning of an MHC class I gene would make it the first nuclear protein-coding gene studied at the DNA level in this species. Because of the large body of data accumulated on different vertebrates (Jarvi *et al.*, 1999; Kaufman *et al.*, 1999; Westerdahl *et al.*, 1999; Richardson and Westerdahl, 2003; Mesa *et al.*, 2004; Moon *et al.*, 2005; Xia *et al.*, 2005; Alcaide *et al.*, 2008, 2009), several studies have emphasized the potential of MHC genes as valuable molecular markers for assessing the evolutionary and adaptive potential of endangered populations and species in relation to the menace of changed and emerging diseases (*e.g.*, Yuhki and O'Brien, 1990; Hedrick and Parker, 1998; Garrigan and Hedrick, 2001; Wan *et al.*, 2006; Bollmer *et al.*, 2007). It is important to point out that nearly all the above cited studies investigating MHC class I diversity in non-model avian species have done so at the cDNA level. In the present study, we cloned and sequenced the α 1 domain encoded by exon 2 and the α 2 domain encoded by exon 3 of the bar-headed goose MHC class I heavy chain. We identified at least four MHC class I genes in one individual, which share great similarity with the MHC class I of the goose and duck. These studies will ultimately improve our understanding of the immune response to pathogens in the bar-headed goose and of avian MHC evolution.

Liver samples from six dead bar-headed geese were collected randomly at the same site of Lake Qinghaihu, Qinghai province, China. The samples were stored at -80 °C at the National Research Center for Wildlife-Borne Diseases, Key Laboratory of Animal Ecology and Conservation Biology, Institute of Zoology, Chinese Academy of Sciences. Genomic DNA (gDNA) was extracted from a piece of liver tissue using the TIANamp Genomic DNA Kit. The first pair of conserved regions of the MHC class I primers designed for the amplification was based on the MHC class I sequence of chicken, duck, goose, turkey and quail. BHGMF 5'-GGTTGTGTTACAGGGTCTCA-3' and BHGMR 5'-TAGCCCTTCTCCTTCTTCCCT-3' were designed to amplify partial MHC class I heavy-chain sequences, which included the expected size of the fragment amplified by using BHGMF and BHGMR primers. Another set of primers was designed based on the sequences obtained from the first primers and corresponding sequences from goose and duck. GMF 5'-AGACGGGTGGGGGTCCTGGA-3' and AIMR 5'-ACTTCCTCTTGGTGATTTGTGCCC-3' were designed to amplify partial upstream MHC class I sequences. We used MHC class I sequences from duck (Moon *et al.*, 2005) and goose (Xia *et al.*, 2005) to identify the exons 2 and 3 of the bar-headed goose MHC class I partial sequences which were amplified in our experiments. These two pairs of primers were designed by software Primer Premier 5.0 (PREMIER Biosoft International, Palo Alto, CA)

PCR was performed in a total volume of 30 μL, containing 20-50 ng of gDNA, 0.5 μM of each primer, and 15 μL of 2PCR Taqmix. Cycling conditions were: 94 °C for 5 min, then 30 cycles at 94 °C for 50 s, 58 °C for 50 s, and 72 °C for 1 min; and a final step at 72 °C for 7 min. The PCR products were analyzed on 1% agarose gel stained with ethidium bromide. Purified PCR products were ligated to the pMD18-T vector and then transformed into *E. coli* DH5α-competent cells. Positive clones were identified by insert release after *Bam*HI and *Hind*III restriction digestion as well as colony PCR and restriction analysis of the PCR products and then sequenced using primer M13. We amplified and cloned DNA from each individual at least three times, and we sequenced at least six positive clones from each individual. The sequences of the six bar-headed geese were submitted to BLAST queries, to ensure that the proper sequences had been amplified. Because Taq polymerase has a significant error rate, nucleotide sequences that varied by only a single base were assumed to represent PCR artifacts and were not identified as unique alleles, unless they were confirmed in a certain number of animals or in separate PCR assays. The nucleotide sequences reported in this article were submitted to GenBank (accession numbers FJ606105 to FJ606113) (Table 1).

Alignments of the bar-headed goose MHC class I gene sequences were obtained by the CLUSTAL X program (1.8) (Thompson *et al.*, 1997) and refined by visual inspection. Manual adjustments of protein-coding nucleotide sequence alignments were facilitated through translation to amino acid sequences. The MEGA 3.1 software (Kumar *et al.*, 2004) was used to estimate the rate of nonsynonymous (*d*_*N*_) and synonymous (*d*_*S*_) substitutions, as described by Nei and Gojobori (1986). *Anin-A1* and *Anin-A2* PBR amino acid sequences were imported into the NCBI Molecular Modeling Database to compare the tertiary structure with the structure of human MHC class I (*HLA-A2*). A phylogenetic tree was constructed with MEGA 3.1 from the predicted amino acids plus previously published vertebrate sequences, using the Neighbor-Joining (NJ) method.

In the six bar-headed geese studied, we confirmed the presence of five sequences of exon 2: Anin-A1*01, Anin-A1*02, Anin-A1*03, Anin-A1*04, and Anin-A1*05. By this nomenclature, “*A1*” refers to the MHC class I α 1 chain gene, while the first two letters of *Anin* are derived from the genus name *Anser* and the last two from the species name *indicus* (Klein *et al.*, 1990; Ellis *et al.*, 2006). Two of these sequences, Anin-A1*01 and Anin-A1*03, were found in all six animals, while Anin-A1*02, Anin-A1*04 and Anin-A1*05 were present in only one of them (Table 1). The amino acid translations deduced from these five sequences produced five unique protein sequences, aligned in Figure 1a. A comparison to the GenBank database revealed that the bar-headed goose MHC class I exon 2 sequences shared great nucleotide similarity with goose and duck MHC class I (79.1% to 91.6%), while their amino acid similarity ranged from 65.9% to 86.3% with goose MHC class I and 65.9% to 73.8% with duck MHC class I, respectively.

**Figure 1:**
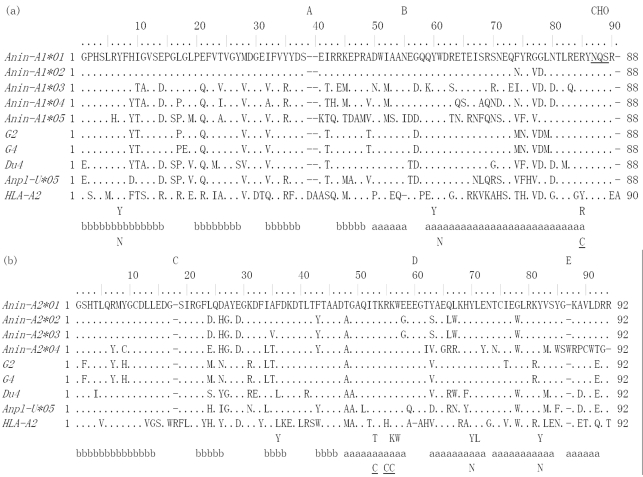
Alignment of the predicted MHC class I (a) α 1 and (b) α 2 domain amino acid sequence of bar-headed goose, goose (*G2*, AY387649; *G4*, AY387651), duck (*Du4*, AB115244; Anpl-U*05, AY294419), and human (*HLA-A2*, K02883). The first amino acid of the α 1 and α 2 domain is considered as position 1 separately. Amino acid identity with Anin-A1*01 and Anin-A2*01 is shown by dot. Conserved amino acid residues involved in binding the peptide-terminal main chain atoms are indicated below the alignment (Moon *et al.*, 2005). aaaaa alpha helix; bbbbb beta strand; N or C residues binding peptide NH2- or COOH- termini; CHO glycosylation site; A, B, C, D, E observed indels between the bar-headed goose sequences and *HLA-A2*. The N-linked glycosylation sites are underlined.

Positive clones sequenced from the six bar-headed geese studied yielded four distinct nucleotide sequences. An alignment of the amino acid translation of the four unique bar-headed goose sequences Anin-A2*01, Anin-A2*02, Anin-A2*03 and Anin-A2*04 is shown in Figure 1b. “*A2*” refers to the MHC class I α 2 chain gene. A comparison to the GenBank database revealed high nucleotide similarity of bar-headed goose exon 3 sequences to goose and duck MHC-I (85.8% to 90.5%) and a high amino acid similarity between them and the MHC class I of goose and duck, which ranged from 86.9% (goose) to 82.6% (duck).

The amino acid sequences deduced for the bar-headed goose MHC class I exons 2 and 3 correspond, respectively, to amino acid positions 26-113 in the α 1 domain and 114-205 in the α 2 domain of the duck MHC class I (Mesa *et al.*, 2004). To determine which genes could be involved in antigen presentation, we examined the amino acid sequences for hallmarks of classical MHC class I genes. Among residues predicted to be involved in peptide anchoring, all were conserved in most loci (Figure 1). The Y35 sequence (Figure 1b) is replaced by phenylalanine in all six bar-headed goose genes, as previously described for the duck (Moon *et al.*, 2005). In order to look for functional diversity within the bar-headed goose MHC class I, rates of synonymous and nonsynonymous substitutions were calculated at probable pocket residues and at the codons that are nonpocket residues for the species studied (Table 2). Nonsynonymous rates were higher at the pocket residues than at the nonpockets in the α 1 domain. At the pocket residues of the α 1 domain, *d*_*N*_ was higher than *d*_*S*_ for any species (*d*_*N*_ /*d*_*S*_ > 1), while at the nonpocket residues, *d*_*N*_ was not higher than *d*_*S*_ in any of the three species.

A phylogenetic analysis of bar-headed goose MHC class I sequences was conducted using the NJ method. Bootstrap values supported bar-headed goose and goose MHC class I exon 2 and exon 3 sequences clustering within the anseriform clade at the order level, and with anseriforms (goose and duck) at the family level (Figure 2). Bar-headed goose exons 2 and 3 clustered with strong support with anseriforms MHC class I sequences. The position of bar-headed goose MHC class I exon 2 and exon 3 sequences in the same clade with goose and duck, along with long branch lengths (Figure. 2), suggests an ancient history of these MHC class I sequences.

**Figure 2:**
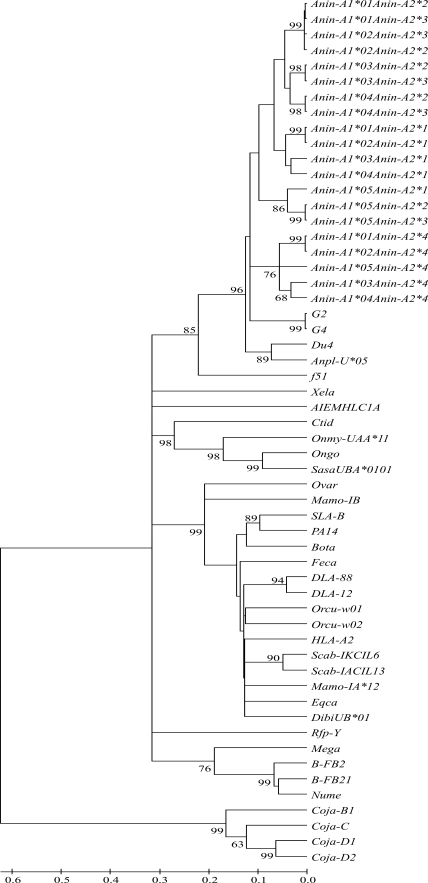
Phylogenetic tree created by the NJ method based on the amino acid sequences of bar-headed goose MHC class I α 1 and α 2 domains and other vertebrates. Only bootstrap levels exceeding 60% are shown (as a percentage) and are based on 1,000 replications. The sequence sources are as follow: goose (*G2,* AY387649; *G4*, AY387651), duck (*Du4*, AB115244; Anpl-U*05, AY294419), human (*HLA-A2*, K02883), rainbow trout (Onmy-UAA*11, AB012064), chicken (*B-FB2*, AF013492; *Rfp-Y*, AF218783; *B-FB21,* AF013493), turkey (*Mega*, DQ993255), helmeted guineafowl (*Nume*, EF643463), grass carp (*Ctid*, AB109779), African clawed frog (*Xela*, L20733), dog (*DLA-88*, U55028; *DLA-12*, U55026), pig (*SLA-B*, AY135599; *PA14*, AF014001), *Ameiva ameiva* (*AIEMHLC1A*, M81094), pink salmon (*Ongo*, D58386), Atlantic salmon (SasaUBA*0101, AF504019), horse (*Eqca*, X71809), domestic cat (*Feca*, U07674), *Diceros bicornis minor* (DibiUB*01, AF055347), sheep (*Ovar*, M34676), cattle (*Bota*, Y09208), rabbit (*Orcu-w01*, K02442; *Orcu-w02*, K02441), woodchuck (Mamo-IA*12, AF146092; *Mamo-IB*, AF201912), tassel-eared squirrel (*Scab-IKCIL6*, M97618; *Scab-IACIL13*, M97617), Japanese quail (*Coja-C*, BAA83671; *Coja-D1*, BAC82515; *Coja-D2*, BAC82516; *Coja-B1*, BAC82519), Florida sandhill crane (f51, AF033106).

To our best knowledge, this is the first report on the isolation of polymorphism patterns in classical MHC class I genes of the bar-headed goose and one of the very few studies of class I gene structure in non-model avian species. Although we did not perform gene expression analyses in this study, other studies have generally observed a correlation between signatures for balancing selection and level of expression of MHC genes (Zoorob *et al.*, 1990; Jacob *et al.*, 2000).

In conclusion, we present here the first MHC class I sequences of the bar-headed goose, a worldwide migrant bird. The molecular methods and sequence data collected in this paper should contribute to a better understanding of the evolutionary significance and conservation implications of the MHC in this species. Moreover, the group-specific primers designed for this study targeted highly conserved regions across several kinds of bird MHC class I genes, and therefore similar fragments of other avian groups are likely to be cross-amplified successfully. Given that MHC genes can decisively determine virus and parasite resistance (Hedrick, 2001), this study may also aid in the preservation of the genetic diversity of the bar-headed goose.

## Figures and Tables

**Table 1:** The sequences of exon 2 and exon 3 in the six bar-headed geese studied.

Exon 2	GenBank accession number	Animal ID	Exon 3	GenBank accession number	Animal ID
Anin-A1*01	FJ606105	A01, A02, A03, A04, A05, A06	Anin-A2*01	FJ606110	A01, A02, A03, A05, A06
Anin-A1*02	FJ606106	A06	Anin-A2*02	FJ606111	A01, A02, A04, A05, A06
Anin-A1*03	FJ606107	A01, A02, A03, A04, A05, A06	Anin-A2*03	FJ606112	A03, A04
Anin-A1*04	FJ606108	A01	Anin-A2*04	FJ606113	A01
Anin-A1*05	FJ606109	A06			

**Table 2:** Comparison of synonymous (*d*_*S*_) and nonsynonymous (*d*_*N*_) substitution rates in exon 2 and exon 3 (encoding α 1 and α 2 domains separately) of MHC class I heavy chain among three Anseriformes birds: goose, duck and bar-headed goose (our study).

Species		Exon 2				Exon 3		
		*d*_*N*_ ± SE	*d*_*S*_ ± SE	*d*_*N*_ / *d*_*S*_		*d*_*N*_ ± SE	*d*_*S*_ ± SE	*d*_*N*_ / *d*_*S*_
\=tbody Goose (*Anser cygnoides*)^a^	Nonpockets	0.0989 ± 0.0176	0.108 ± 0.0175	0.914		0.0648 ± 0.0115	0.140 ± 0.0317	0.463
	Pockets	0.277 ± 0.0503	0.168 ± 0.0422	1.64		0.208 ± 0.0477	0.320 ± 0.0712	0.648
Duck (*Anas platyrhynchos*)^b^	Nonpockets	0.0692 ± 0.00649	0.127 ± 0.0117	0.547		0.0776 ± 0.00545	0.0955 ± 0.00955	0.813
	Pockets	0.287 ± 0.0361	0.184 ± 0.0370	1.56		0.197 ± 0.0233	0.056 ± 0.0199	3.53
Bar-headed goose (*Anser indicus*)	Nonpockets	0.103 ± 0.0210	0.147 ± 0.0227	0.697		0.0347 ± 0.0144	0.0867 ± 0.0433	0.4
	Pockets	0.338 ± 0.0578	0.234 ± 0.0426	1.44		0.0987 ± 0.0493	0.245 ± 0.123	0.402

Rates were calculated separately for the codons making up the nonpockets and the codons making up the pockets (Xia *et al.*, 2005). ^a^Calculated using sequences from Xia *et al.* (2005). ^b^Calculated using sequences from Mesa *et al.* (2004), Xia *et al.* (2004), Moon *et al.* (2005).
